# Research in Pregnant Subjects: Increasingly Important, but Challenging

**DOI:** 10.31486/toj.19.0077

**Published:** 2020

**Authors:** Joseph R. Biggio

**Affiliations:** System Co-Chair of Women's Services, Ochsner Baptist, New Orleans, LA and Clinical Professor of Obstetrics and Gynecology, The University of Queensland Faculty of Medicine, Ochsner Clinical School, New Orleans, LA

**Keywords:** *Common Rule*, *pregnancy*, *research*, *vulnerable populations*

## Abstract

**Background:** The number of pregnant women with medical comorbidities continues to increase. A large proportion of pregnant women are exposed to medications during pregnancy, but only a fraction of the medications used have been investigated during pregnancy with regard to benefits, risks, and doses.

**Methods:** This article includes a review of potential deterrents and barriers to pregnant women enrolling in clinical research studies and the federal regulations governing enrollment of pregnant women in research.

**Results:** Research in pregnant women has been hampered by concerns for liability, the complex physiology of pregnancy with changes related to stage of pregnancy, and federal regulations that deemed pregnant women a vulnerable population. While recent revisions to federal regulations have removed pregnant women from the classification of vulnerable population, regulations regarding consent requirements still limit women's ability to decide on participation in clinical trials. The Department of Health and Human Services established the Task Force on Research Specific to Pregnant Women and Lactating Women to help identify and reduce these barriers.

**Conclusion:** While recognition of the need for more scientific knowledge on the effects of medications and other interventions in pregnancy is widespread, a number of barriers that hinder enrollment of pregnant women in clinical trials remain.

## INTRODUCTION

In the United States each year, more than 6 million pregnancies and approximately 4 million births occur.^1^ Up to 90% of women are estimated to be exposed to at least one medication during pregnancy, and at least 60% of women who ultimately give birth take one or more medications to treat a chronic medical condition or a condition that arises during the pregnancy.^2,3^ Among the most common medical conditions for which women take medications during pregnancy are hypertension, diabetes, mental health disorders, and autoimmune conditions. Underlying chronic hypertension affects approximately 5% to 10% of the obstetric population, while diabetes, either pregestational or occurring during pregnancy, affects up to 10% of pregnant women. Because of the paucity of information on risks of medication exposure in pregnancy, nearly 50% of women are estimated to use an agent that has little information on its risk during pregnancy or in which animal data suggest the possibility of adverse human effect.^2,3^

A disproportionately small number of the medications currently on the market have been investigated during pregnancy. Of medications introduced to the market between 1980 and 2000, 91% had undetermined fetal effects, 3% had some known fetal risk, and only 6% had no known fetal risks.^4^ Very few medications on the market have specific indications for use during pregnancy or specific information about dosing in pregnancy. Further, many medications on the market with declared information on risks to a developing fetus are based on toxicity and teratology studies in animals that may not be predictive of human effects.^4-6^

## CLINICAL TRIALS EXCLUSION AND LIABILITY CONCERNS

While the underlying reasons for the dearth of information on drug effects on the developing fetus are multifaceted, the primary explanation for the lack of information is the systematic exclusion of pregnant women from clinical trials. Although potential risks are the typical reason for excluding pregnant women, multiple women's health advocates have pointed out that without adequate information on the use of therapeutic agents during pregnancy, it is not possible to make an informed decision about the risk-benefit analysis, and the use of untested agents may ultimately expose far more women and their fetuses to potential harm than would be encountered with a well-designed clinical trial.^5-7^ In the absence of pregnancy-specific data on medication dosing, typical doses used for treatment in a nonpregnant individual when used in a pregnant women may be insufficient for maternal benefit but still have fetal risk.

The National Institutes of Health Office of Research on Women's Health (ORWH) was established in 1990 to address the overall lack of systematic inclusion of women in clinical research. When the Code of Federal Regulations governing human subjects research (45 CFR §46) was originally established, there was a presumptive exclusion of women from clinical research, largely because of the potential concern regarding pregnancy and inadvertent fetal exposure as the stories of thalidomide and diethylstilbestrol exposure were still prominent in public memory. The desire to prevent other tragedies like these led to the categorization of pregnant women as a vulnerable population.^8^ The vulnerable classification was historically applied to populations that were considered to be unable to be autonomous agents or whose voluntariness might be compromised, such as children, prisoners, and those with diminished mental capacity.^9,10^

While the ORWH was established with a charter to address sex disparity in scientific knowledge in all fields of medicine, issues surrounding the lack of data on medication use in pregnancy, as well as medical comorbidities overall, have become increasingly apparent and warrant additional attention. In 1994, the Institute of Medicine issued a report stating that pregnant women should be presumed eligible for participation in clinical trials and should be excluded only if the trial offered no prospect of medical benefit to the pregnant woman or if the trial involved potential risk of significant harm to the fetus, either known or plausible.^11^ Despite this report, little change was made to federal regulations, and the number of women involved in clinical trials—other than those addressing specific pregnancy issues—only minimally increased.^5,12,13^

## PHYSIOLOGY OF PREGNANCY

Although federal regulations and liability concerns have been major factors limiting inclusion of pregnant women in clinical research, the complex physiology of pregnancy has also been a deterrent.^14^ A number of significant physiologic changes occur during pregnancy that can impact drug metabolism and action. The effective volume of distribution of pharmacologic agents is altered significantly by the expansion of plasma volume, typically 50% to 60%, during pregnancy.^15^ Most pregnant women develop some degree of hypoproteinemia as gestation advances that alters free drug concentration and the potential therapeutic window.^16^ Further, both absorption and clearance of pharmacologic agents are altered. Changes in gastrointestinal motility and gastric acidity affect rates of drug absorption depending on where a particular agent is absorbed. Increases in the glomerular filtration rate and hepatic enzyme activity, stimulated by hormonal effects, result in more rapid clearance of many pharmacologic agents.^15^ All of these factors, as well as the fact that these physiologic changes evolve as pregnancy advances, combine to potentially alter the pharmacokinetics and pharmacodynamics of drugs during pregnancy.^16^ These potential contributors to variations in drug effect make investigation of therapeutics during pregnancy not only more difficult, but also more expensive, leading many pharmaceutical companies and investigators to exclude or minimize the involvement of pregnant women in research.

## FEDERAL REGULATIONS

Much of the concern regarding liability issues with research in pregnancy centers around the historic classification of women as a vulnerable population. The vague and restrictive wording of the federal regulations, with variable interpretation by both local institutional review boards and government agencies, contributes to liability concerns.^5-7^ While historic events involving congenital anomalies associated with the use of medications have led to considerable liability concerns, more recent data on various medications in pregnancy, albeit limited, have not replicated such experiences, especially when appropriate animal models and pregnancy registries have been utilized.^17,18^ And, as noted earlier, the cumulative risk to society is postulated to be lower with scientifically rigorous, carefully monitored clinical studies than with off-label or poorly informed use of medications as often happens in modern obstetric practice.^5-7,19^

In 2001, the Department of Health and Human Services revised Subpart B of the Code of Federal Regulations—known as the Common Rule—to state that pregnant women and their fetuses could be included in research if specific criteria were met.^20^ While the changes were intended to create a more inclusive approach, pregnant women were still often excluded from clinical research trials because of liability concerns, not only associated with fetal risk but also with meeting the necessary criteria for this vulnerable group of study participants for whom the federal regulations required special protections. The changes failed to create a presumption that pregnant women should be included in research.

In 2010, the ORWH held a scientific forum to begin to address some of the ethical and recruitment challenges associated with conducting clinical research in pregnant women. This meeting resulted in the development of a research agenda focused on expanding the knowledge base of medication use in pregnancy and a road map to begin to address ethical and liability concerns regarding research in pregnancy.^14^

One of the discussion points arising from this meeting revolved around the classification of pregnant women as a vulnerable population. As defined in the Common Rule, vulnerability means “vulnerable to coercion and undue influence, in recognition that coercion or undue influence refers to the ability to make an informed decision about participating in research*.*”^12^ Using this definition, many argued that modern principles of medical ethics do not justify such classification because a pregnant woman should have the capacity to decide for herself whether or not to participate in research, as well as the capacity to protect the interests of the fetus. Further, because maternal benefit often results in fetal benefit by either improving overall maternal health or allowing pregnancy prolongation, the capability of the woman to decide is even more paramount.^3,5,6,11,14,21-25^ Some have posited that the only reason pregnant women should be considered a vulnerable population is because the systematic exclusion of pregnant women from clinical research has rendered them vulnerable to the inability to make informed decisions about medical therapies because of the a lack of high-quality evidence.^25,26^ In 2016, the American College of Obstetricians and Gynecologists (ACOG) published a document stating that (1) pregnant women have similar capacity for autonomy as nonpregnant women, (2) inclusion is in accordance with the ethical principle of justice after disclosure of all appropriate risks, (3) proscriptive contraceptive requirements for participation reduce patient autonomy, and (4) partner consent is unwarranted and ethically unjustified as it infringes on maternal autonomy.^21^ With this background, ACOG challenged that pregnant women should no longer be considered vulnerable, but should instead be considered scientifically complex because of the associated ethical and physiologic complexities.

With momentum building to address these issues, Congress passed the 21^st^ Century Cures Act in 2016 that directed the Secretary of Health and Human Services to establish the Task Force on Research Specific to Pregnant Women and Lactating Women.^27^ The task force was charged with identifying gaps in knowledge of safe and effective therapies in pregnant and lactating women and advising the secretary on how to best address those deficiencies. The summary report identified a number of barriers and challenges to research in pregnant women, including regulatory, liability, resource, and investigator challenges.^13^ A prominent point of discussion at early meetings focused on classification of pregnant women as a vulnerable population and potential changes to the Common Rule. As a result of the growing recognition of the need for better scientific knowledge regarding medical treatments during pregnancy, the Office for Human Research Protections changed the Common Rule to remove pregnant women as a vulnerable population.

The revised Common Rule maintained and did not, however, change the criteria, originally specified in 2001, that must be fulfilled for pregnant women and their fetuses to be included in clinical research (Table). The first three requirements endeavor to ensure that the risk-benefit analysis is considered and that research design focuses on minimizing potential risk. Prior to initiation of a clinical trial in pregnant women, the requirement of data in pregnant animals and nonpregnant women provides insight into the potential safety of the agent under study and helps gauge the degree of risk and putative benefit that might be recognized. These data inform decisions regarding the “prospect of direct benefit for the woman or the fetus” and help to determine if the risk is greater than minimal. These determinations of direct benefit and minimal risk are critical, as they form the foundation around which many of the additional requirements are framed.

**Table.** Code of Federal Regulations Requirements for Inclusion of Pregnant Women and Their Fetuses in Clinical Research

a) Where scientifically appropriate, **preclinical studies**, including studies on pregnant animals, and clinical studies, including studies on nonpregnant women, have been conducted and provide data for assessing potential risks to pregnant women and fetuses.
b) The risk to the fetus is caused solely by interventions or procedures that hold out the **prospect of direct benefit for the woman or the fetus**; or, if there is no such prospect of benefit, **the risk to the fetus is not greater than minimal** and the purpose of the research is the development of important biomedical knowledge which cannot be obtained by any other means.
c) Any **risk is the least possible** for achieving the objectives of the research.
d) If the research holds out the **prospect of direct benefit to the pregnant woman, to the pregnant woman and the fetus, or no prospect** of benefit for the woman or the fetus when **risk to the fetus is not greater than minimal** and the biomedical knowledge cannot be obtained by any other means, **her consent is obtained**.
e) If the research holds out the **prospect of direct benefit solely to the fetus** then the consent of the pregnant woman and the father is obtained, except that the father's consent need not be obtained if he is unable to consent because of unavailability, incompetence, or temporary incapacity or the pregnancy resulted from rape or incest.
f) Each individual providing consent is fully informed regarding the reasonably foreseeable impact of the research on the fetus or neonate.
g) For children who are pregnant, assent and permission are obtained in accord with the provisions of Subpart D (Research Protection for Children).
h) **No inducements will be offered to terminate** a pregnancy.
i) Individuals engaged in the research will have no part in any decisions as to the timing, method, or procedures used to terminate a pregnancy.
j) Individuals engaged in the research will have no part in determining the viability of a neonate.

Note: Text is adapted from 45 CFR 46 Subpart B §46.204.^12^

For research with a potential direct benefit to the pregnant woman or to both the woman and her fetus, only consent from the pregnant woman is required. Similarly, if the research has no prospect of direct benefit to the pregnant woman or the fetus and the risk to the fetus is no more than minimal, research that will generate important medical knowledge can be conducted with the consent of the pregnant woman. In contrast, if the research has potential direct benefit only to the fetus, consent of both the mother and the father is required unless he is not available, he is incompetent, or the pregnancy resulted from rape or incest. This requirement for consent from both the mother and the father in the setting of pregnancy is in contradistinction from the consent requirement in pediatric research where consent of only a single parent is required when the research has no potential direct benefit to the child. The rationale for this discrepancy has been questioned as it raises the question of why a woman has capacity to consent for her child after birth but lacks such capacity prior to birth.^13^ The final requirements focus on avoiding potential risk and conflicts associated with pregnancy termination and determination of viability.

A key point of contention for application of the Common Rule requirements revolves around the definitions of minimal risk and direct benefit.^28^ Per 45 CFR §46.102.i, minimal risk is defined as “the probability and magnitude of harm or discomfort anticipated in the research are not greater in and of themselves than those ordinarily encountered in daily life or during the performance of routine physical or psychological examinations or tests.”^12^ Applying this standard to the fetus is problematic. What constitutes ordinary daily risk is highly variable—especially in the setting of a complex medical condition that places the fetus at risk every day—and subject to considerable variability in interpretation. Similar challenges arise when attempting to apply the prospect of benefit to the mother or fetus criterion and lead to variations in interpretation of how much benefit and how high a prospect is required to meet the standard.

Originally scheduled for implementation in 2018, the modified Common Rule did not go into effect until 2019, so the extent to which this change will increase the number of pregnant women involved in clinical research and augment our knowledge base is unknown. At later meetings of the Task Force on Research Specific to Pregnant Women and Lactating Women, task force members expressed that removal of the vulnerable population classification and required special protections would hopefully signal the desire for more research in pregnant women to local institutional review boards, other regulatory agencies, and investigators.^13^ However, because other criteria specified in Subpart B of the Common Rule were not modified, concern remains about whether the above change will be enough to stimulate increased research in pregnant women and expand scientific knowledge regarding medication and other interventions in pregnancy.

## CONCLUSION

With the increasing number of pregnant women with comorbid medical conditions, the need to expand our scientific knowledge and ensure participation of pregnant women in research is critical. Enhanced knowledge is required to better understand the risks and benefits of treatment to the mother and the fetus. The traditional approach to enrollment of pregnant women in research has been one of exclusion because of liability concerns and federal regulations subject to variable interpretations. Recent changes in federal regulation have tried to encourage a paradigm shift toward inclusion, but whether these changes will be enough to appreciably increase the number of pregnant women enrolled in clinical trials is unclear.

## Notes

This article meets the Accreditation Council for Graduate Medical Education and the American Board of Medical Specialties Maintenance of Certification competencies for Patient Care, Medical Knowledge, and Systems-Based Practice.
